# Assessing Obstructive Sleep Apnea Severity During Wakefulness via Tracheal Breathing Sound Analysis

**DOI:** 10.3390/s25206280

**Published:** 2025-10-10

**Authors:** Ali Mohammad Alqudah, Zahra Moussavi

**Affiliations:** 1 Biomedical Engineering Program, University of Manitoba, Winnipeg, MB R3T 5V6, Canada; alqudaha@myumanitoba.ca; 2 Department of Electrical and Computer Engineering, University of Manitoba, Winnipeg, MB R3T 5V6, Canada

**Keywords:** obstructive sleep apnea, OSA severity prediction, tracheal breathing sounds, wakefulness screening

## Abstract

**Highlights:**

**What are the main findings?**

- This study demonstrates a significant correlation between tracheal breathing sounds (TBS) recorded during wakefulness, anthropometric features, and the apnea–hypopnea index (AHI).
- A machine learning model trained on these features can form the basis of classifications of OSA severity in standard clinics.
- Categories (Non-, Mild-, Moderate-, and Severe-OSA) are formed without the need for sleep-based recordings.

**What is the implication of the main finding?**

- The proposed method enables the rapid, low-cost, and accessible estimation of OSA severity using brief, wakefulness-based TBS and basic anthropometric data.
- This approach can serve as a reliable screening and triage tool in clinical settings, helping reduce perioperative risks by informing earlier intervention and referral for full diagnosis.

**Abstract:**

Obstructive sleep apnea (OSA) is a commonly underdiagnosed condition that not only increases the risk of accidents but also significantly contributes to a wide range of health complications, including heightened perioperative morbidity and mortality risks during surgeries under general anesthesia. Polysomnography (PSG), which is the diagnostic gold standard, is costly, requires skilled technicians, is time-consuming, and is not always accessible. This study presents a fast, objective, and non-invasive method for detecting OSA severity by analyzing tracheal breathing sounds (TBS) recorded during wakefulness in supine position. Features were extracted from six binary (1-vs-1) severity comparisons—Non-OSA, Mild, Moderate, and Severe—and combined with anthropometric characteristics for classification. The data of 199 subjects (74 Non-OSA, 35 Mild, 50 Moderate, and 40 Severe) were analyzed, the data of 169 and 30 was used for training and blind testing, respectively, and the training dataset was shuffled 10 times to avoid any bias during training. Multiple machine learning models were evaluated, and the best-performing model for each was saved. Across six experimental models comparing OSA severity levels, the most balanced performance was achieved by the Base Model of Non-OSA vs. Severe-OSA using the support vector machine algorithm, with 88.2% accuracy, 83.3% sensitivity, and 90.9% specificity. While Random Forests in the Base Model of Non-OSA vs. Mild-OSA achieved 100% sensitivity, its accuracy was lower (81.2%). The results confirm the reliability and robustness of the proposed approach, providing a basis for OSA severity screening in under 10 min during wakefulness.

## 1. Introduction

Obstructive sleep apnea (OSA) is a common but underdiagnosed sleep-related breathing disorder, affecting nearly 20% of adults in Canada and the United States [1,2]. Alarmingly, up to 90% of cases remain undiagnosed, with affected individuals often unaware of their condition or left untreated [3]. The absence of diagnosis and treatment carries substantial healthcare and economic consequences; in the United States, the added direct and indirect costs of untreated OSA are estimated at USD 65–165 billion annually [4,5,6].

OSA accounts for more than 75% of sleep apnea cases and is caused by recurrent collapse of the upper airway during sleep, leading to complete (apnea) or partial (hypopnea) airflow obstruction [7,8]. Events lasting longer than 10 s with an oxygen desaturation of at least 3% are classified as apneas or hypopneas [9,10]. Clinically, OSA presents with both nighttime symptoms (e.g., loud snoring, gasping, frequent awakenings) and daytime symptoms (e.g., fatigue, morning headaches, depression, excessive sleepiness) [11]. The severity of OSA is defined by the apnea–hypopnea index (AHI), with thresholds of 0–5 (Non-OSA), 5–15 (Mild), 15–30 (Moderate), and >30 (Severe) events per hour [8,12]. The diagnostic gold standard is overnight polysomnography (PSG), but PSG is costly, resource-intensive, and often associated with waiting times of 3–12 months [13]. Portable monitors offer a more accessible alternative, but they still require overnight use and physician confirmation [14,15].

Identifying OSA severity prior to surgery is particularly important for perioperative risk stratification, as undiagnosed OSA significantly increases the risk of adverse outcomes [4,14,15]. Current alternatives to PSG often rely on screening questionnaires (e.g., STOP-Bang, Berlin) and anthropometric measures (e.g., age, BMI, gender), which are highly sensitive but limited by low specificity (~10%) [16,17]. Given the limitations of overnight PSG and questionnaire-based tools, there is a pressing need for objective, wakefulness-based methods that can directly assess OSA severity.

Our group and others have pioneered the use of tracheal breathing sounds (TBS) recorded during wakefulness to screen for OSA in a binary manner with high accuracy [18,19,20,21,22,23,24,25]. However, existing studies have not addressed OSA severity classification, despite its critical role in perioperative planning. The risk of complications varies significantly across severity levels, with severe OSA associated with increased rates of respiratory failure and cardiovascular events [8,26,27]. Accurate severity detection could therefore guide anesthetic management, postoperative monitoring, and preoperative interventions, ultimately improving surgical safety.

In this study, we introduce a novel algorithm for multi-class OSA severity classification during wakefulness, using features extracted from TBS combined with anthropometric data. We further interpret the extracted features from both physiological and feature-importance perspectives, laying the groundwork for a non-invasive and practical screening framework.

## 2. Literature Review of Tracheal Breathing Sounds Analysis

As this research is based on tracheal breathing sound (TBS) analysis during wakefulness, it is important to review prior studies in this field. Spectral and bispectrum features of the TBS have been the focus of several studies to classify OSA and non-OSA groups [19,20,21,24,28]. Early works applied power spectral density, kurtosis, and fractal dimension of tracheal sounds during wakefulness for OSA severity classification, achieving up to 91.7% accuracy in distinguishing severe OSA (AHI > 30) from non-OSA (AHI < 5) using LDA and QDA classifiers [21]. Combining anthropometric and TBS features with support vector machines (SVMs) yielded 83.9% accuracy in detecting OSA at an AHI ≥ 10 [20]. A subsequent ensemble framework based on subgroup-specific anthropometric models improved robustness, achieving 81.4% accuracy, 80.9% sensitivity, and 82.1% specificity for detecting OSA at the clinically relevant threshold of AHI > 15 [19]. More recently, combining spectral and bispectrum features with anthropometric data enabled prediction of PSG-derived parameters such as arousal index and mean SpO_2_ with 88.8% accuracy in blind testing [29].

Machine learning has further advanced this field. Logistic regression with LASSO-based feature selection achieved 79.3% ± 6.1% accuracy in blind testing [23]. Comparative studies later showed that Random Forest (RF) outperformed regularized logistic regression in both sensitivity and specificity for OSA detection using TBS and anthropometric data, at thresholds of AHI < 5 (Non-OSA), 5 ≤ AHI < 15 (Mild OSA), and AHI ≥ 15 (Moderate-to-Severe OSA) [22]. Beyond spectral measures, formant features extracted from tracheal breathing sounds showed significant group differences, with a sensitivity of 88.9% and specificity of 84.6%, when combined with anthropometrics [25]. Speech-based approaches have also been explored, where a composite system analyzing breathing segments, vowels, and continuous speech achieved 77.1% accuracy for distinguishing OSA at an AHI threshold of 15, offering a complementary alternative to TBS [24].

A recent review has summarized these methodologies, highlighting the strong diagnostic potential of TBS analysis during wakefulness as a cost-effective and accessible screening tool [26]. Advanced acoustic and anthropometric-aware machine learning methods show particular promise, but nearly all studies to date focus on binary classification at thresholds such as AHI ≥ 15 or AHI ≥ 10 versus ≤ 5. Multi-class severity classification (mild, moderate, severe) remains a significant challenge during wakefulness. While previous works have focused mainly on binary OSA detection during wakefulness, our study uniquely advances severity classification by integrating image-based morphological features and SHAP-guided feature selection. To the best of our knowledge, no study has yet addressed this gap. Given the importance of OSA severity detection, especially for perioperative risk stratification, this study proposes a non-invasive, multi-class wakefulness-based framework that could support earlier diagnosis and reduce reliance on overnight sleep assessments.

## 3. Materials and Methods

**Figure 1:**
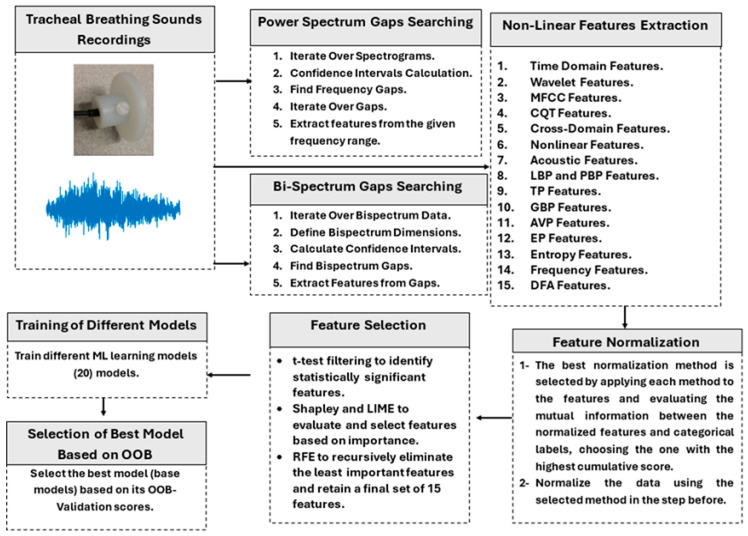
Block diagram of the proposed methodology. The process includes (1) preprocessing of tracheal breathing sounds (segmentation, filtering, normalization), (2) extraction of spectral, temporal, nonlinear, and morphological features, (3) feature selection using *t*-test, SHAP ranking, and RFE, and (4) classifier training with bootstrap aggregation and OOB validation.

The present study aims to classify OSA severity into three classes—Mild, Moderate, and Severe OSA—and include healthy subjects (non-OSA) by utilizing features from different domains and representations. The proposed technique is comprehensively detailed in the following subsections, and Figure 1 shows a block diagram of the proposed methodology.

### 3.1. Tracheal Breathing Sounds Dataset

**Figure 2:**
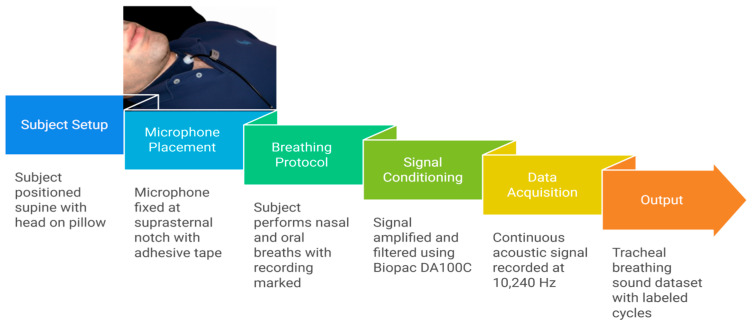
Experimental setup for TBS recordings. A Sony ECM77B condenser microphone was positioned at the suprasternal notch using a 2 mm custom plastic chamber to ensure consistent skin coupling and minimize ambient noise. Signals were sampled at 10,240 Hz using the Biopac DA100C (Biopac, Goleta, CA, USA), while participants were in a supine position during controlled wakefulness breathing maneuvers.

In this work, the dataset used was adopted from our team’s previous works [19]. The data were collected from 199 subjects, and the recording was made while the subjects were awake in a supine position with a pillow. Then, each individual’s TBS were recorded using a Sony ECM-77B, Tokyo, Japan omnidirectional condenser microphone (sensitivity: −52 dB ± 3.5 dB, frequency response: 40 Hz–20 kHz) positioned at the suprasternal notch via a custom 2 mm plastic chamber [20]. This setup minimized ambient noise and ensured consistent skin-to-microphone coupling. A schematic of microphone placement is provided in Figure 2. Then, each subject completed five cycles of deep breathing through the nose with the mouth closed and another five breaths through the mouth while wearing a nasal clip.

In this research, unlike our previous works, the AHI of subjects was grouped into four categories: Non-OSA (n = 109, AHI < 5), Mild-OSA (n = 109, 5 ≤ AHI < 15), Moderate-OSA (n = 109, 15 ≤ AHI < 30), and Severe-OSA (n = 90, AHI ≥ 30). Table 1 presents the total number of subjects in each severity group, along with their corresponding anthropometric data, for the dataset used in this study.

#### 3.1.1. Splitting Dataset for Training and Testing

Then, the data was split once into training and testing sets with ratios of 85% and 15%, respectively. These percentages were chosen to balance model training efficiency and evaluation reliability for the six distinct experiment models, as explained below. The training was repeated 10 times with a shuffled training dataset to avoid any bias during training. The 85% training portion provided enough samples to support the learning needs of both individual experiment models without leading to overfitting. Meanwhile, the 15% testing subset was carefully curated to maintain class balance and preserve the distributional characteristics of the original dataset across key variables, including sex, Mallampati score (MPS), apnea–hypopnea index (AHI), body mass index (BMI), age, and neck circumference (NC). For instance, the AHI averages in the testing datasets closely match those in the overall dataset for each OSA class (e.g., Non-OSA: 1.2 vs. 0.86; Mild: 8.7 vs. 6.7; Moderate: 21.5 vs. 20.7; Severe: 69.5 vs. 80.0), indicating that disease severity is well-represented in the testing data.

Similarly, it was ensured that the distributions of BMI, age, and NC in the testing set fell within one standard deviation of the overall means, reflecting a non-biased sample selection. This stratified approach ensures that the trained model was evaluated on a representative, diverse, and clinically meaningful subset of patients, thereby enhancing the generalizability and robustness of the findings. Moreover, this data split allows for sufficient subgroup representation, even within smaller classes (e.g., Mild-OSA), thereby avoiding skewed model evaluation due to under-sampling or class imbalance. The chosen ratio also aligns with standard medical machine learning studies practices, where datasets are typically limited, and a larger training set can significantly improve model convergence and stability. Table 2 and Table 3 show the distribution of the anthropometric data of the training and testing subjects for one split.

#### 3.1.2. Splitting Dataset for K-Fold

For this problem, the regular stratified K-fold cross-validation was not suitable for this research due to the need to maintain strict stratification across clinically significant anthropometric factors in addition to the severity classes, and to avoid variability in class representation within smaller OSA classes. Therefore, a custom stratified K-fold approach was designed, where the folds were balanced simultaneously across both the OSA severity groups and the key confounding anthropometric thresholds, including age (<50 vs. ≥50), BMI (<35 vs. ≥35), neck circumference (≤40 vs. >40), sex (male vs. female), and Mallampati scores. This ensured that each fold preserved the joint distribution of clinically relevant subgroups, thereby reducing the risk of bias and making the training and evaluation processes more representative of the real-world population’s heterogeneity.

By enforcing these stratification rules, each training and test split captured not only the proportional distribution of OSA severity classes but also the underlying demographic and anatomical risk factors. This level of control was essential for producing generalizable and reliable models, especially in subgroups with limited sample sizes that might otherwise be underrepresented in conventional splitting strategies. Table 4 shows the distribution of subjects’ anthropometric data of the k-fold splits.

### 3.2. Tracheal Breathing Pre-Processing

**Figure 3:**
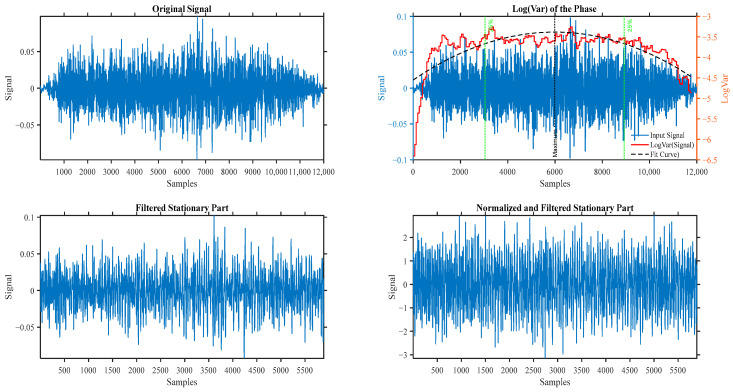
The results of preprocessing techniques on a sample breathing phase.

A series of pre-processing steps was applied to prepare the raw audio recordings for analysis. First, all recorded signals went through a check in the time and frequency domains to check if there was any background noise or vocal noise; then, the signals underwent segmentation into the inspiratory and expiratory phases and the signal to noise ratio (SNR) was calculated between each phase and background to remove any phase with a very low SNR [19,20]. This separation was crucial, as upper-airway obstructions often manifest differently in each respiratory phase, particularly in patients with OSA [20]. The segmentation was achieved using a log(var) of the signal with a thresholding approach to isolate the breath cycles [19,20]. Following segmentation, a 4th-order Butterworth bandpass filter with cutoff frequencies of 75–3000 Hz was applied to reduce the effect of heartbeats, microphone artifacts, muscle motion, 60 Hz harmonics, and ambient noise [19,20]. Finally, all filtered signals were normalized using two methods: through their variance envelope (a smoothed version of the sample moving average) and then using their standard deviation (energy) to eliminate the effect of plausible airflow fluctuation between the breathing cycles [19,20]. Figure 3 shows the results of the preprocessing techniques on a sample breathing phase.

### 3.3. Anthropometric Missing Value Imputation

**Figure A1:**
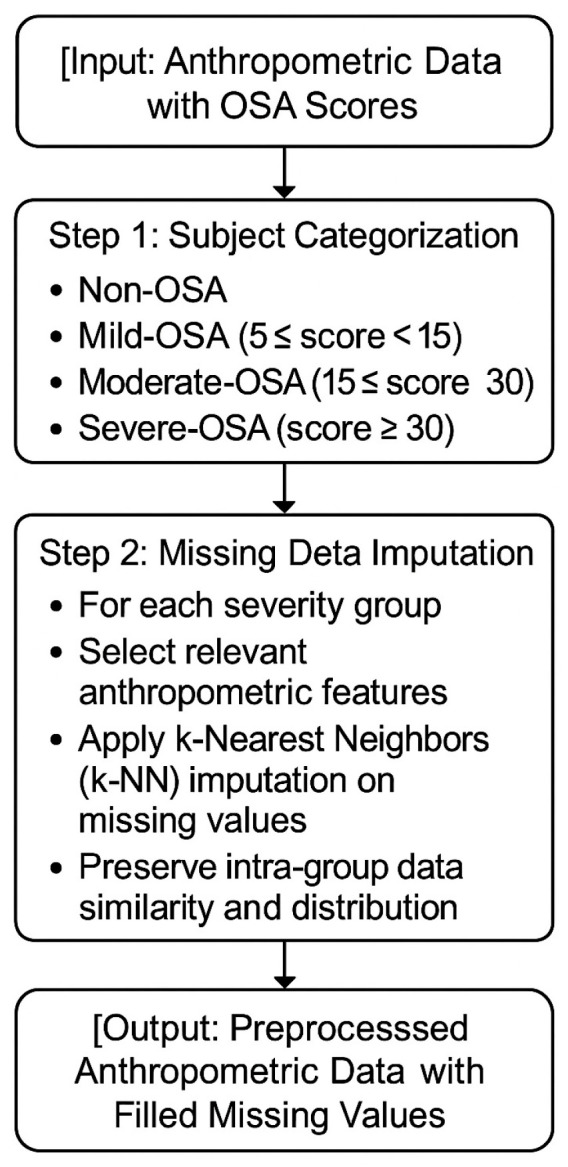
Flow chart of missing anthropometric data filling.

Missing anthropometric values were imputed using a severity-specific k-nearest neighbors (k-NN) method to maintain internal group distributions and minimize bias [30,31]. The full imputation methodology and implementation steps are detailed in Appendix A, Figure A1.

### 3.4. Feature Extractions

**Figure 4:**
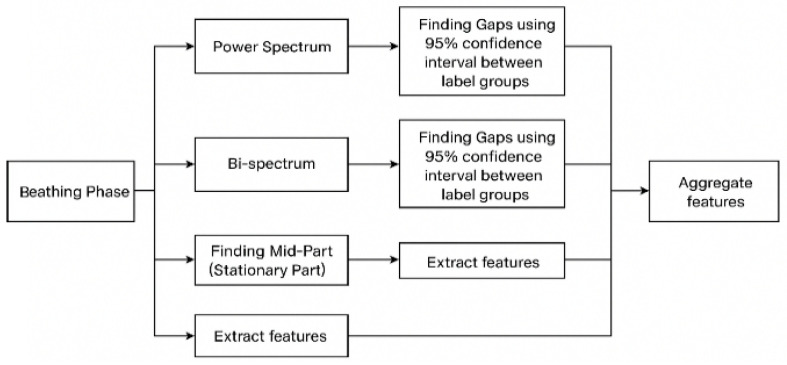
Flow chart of feature extraction from training data.

The feature selection methodology spans multiple analytical domains, including spectral, temporal, nonlinear, and cross-domain analyses, ensuring a holistic and multidimensional representation of linear and nonlinear signal dynamics. The extracted features are grouped and optimized specifically for each base model (binary classifiers). This model-specific feature selection process enables the creation of personalized feature sets that enhance model robustness, improve classification accuracy, and support high-performance modeling for diagnostic and predictive applications. The parameters for power spectrum and bispectrum gaps from confidence intervals are calculated from the training dataset and then applied to the testing dataset. Figure 4 illustrates the steps involved in feature extraction.

Finding gaps in power spectrum and bispectrum using confidence intervals—identifying meaningful deviations in frequency-domain representations, such as the power spectrum and bispectrum, is critical for understanding the underlying dynamics of non-stationary signals and pinpointing the regions to focus on during feature extraction. Traditional spectral analysis often relies on peak detection or energy thresholds, which can overlook subtle but statistically significant features. To enhance this process, we employed confidence interval-based gap detection, allowing for the quantification of spectral features that deviate meaningfully from expected background fluctuations.

For the power spectral density (PSD), we first estimated the mean spectrum across subjects or trials and computed the standard deviation at each frequency bin. Assuming normality in the spectral estimates, a valid approximation under the Central Limit Theorem for large sample sizes, a 95% confidence interval was constructed as follows:

(1) $$CI\left(f\right)=\mu \left(f\right)\pm 1.96\cdot \sigma \left(f\right)$$

where μ(f) and σ(f) represent the mean and standard deviation of spectral power at the frequency f, respectively. Frequencies where the spectral power of a subject or a class-specific average exceeded or fell below this confidence range were marked as spectral “gaps” or “anomalies,” depending on the context. The same principle was extended to the bispectrum, which captures quadratic phase coupling between frequency components and reveals nonlinear interactions not evident in the power spectrum alone. Due to the higher dimensionality and more complex distribution of Bispectrum estimates, we employed a bootstrap resampling method [32] to compute empirical confidence intervals for the Bispectrum magnitude and phase at each frequency pair $\left(f_{1},f_{2}\right)$. This approach avoids Gaussianity assumption, which is often violated in higher-order spectral domains.

**Figure 5:**
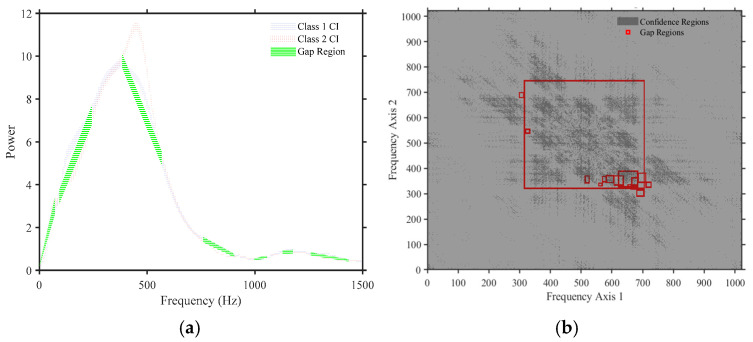
A sample of the detected gaps regions of both PSD and bispectrum where (**a**) shows the PSD detection gaps, highlighted in yellow, using the proposed method; (**b**) shows the regions containing bispectrum detection gaps, highlighted as red boxes, using the proposed method.

Significant Bispectrum gaps were identified where the observed Bispectrum values lay outside the bootstrapped 95% confidence bounds. These gaps may indicate regions of suppressed nonlinear interactions or phase-coupling loss and can be critical in distinguishing pathological signal dynamics from normal states [33,34]. By focusing on confidence-interval-defined deviations, this method provides a statistically principled framework for highlighting underexplored or weakly represented frequency components in both linear and nonlinear spectral representations. Figure 5 shows samples of detected gaps on both PSD and Bispectrum.

Initially, PSD was estimated using Welch’s method to identify frequency bands with significant differences across groups [35]. Within these bands, a set of representative spectral features was extracted, including mean power, spectral centroid, bandwidth, and spectral entropy. To capture nonlinear interactions and higher-order harmonics, bispectrum analysis (higher-order spectral domain) [36] was performed. Features such as bispectrum magnitude, total bispectrum energy, and symmetry metrics were extracted from statistically significant regions identified using confidence intervals.

Time-domain descriptors were also included to account for amplitude dynamics and signal complexity, such as zero-crossing rate, root mean square (RMS), fractal dimension [37], waveform length, shimmer, and jitter [38]. Measures such as Noise-to-Harmonic Ratio (NHR) [39] and correlation coefficients were also used to quantify voice quality and signal regularity. Complementary time-frequency features were extracted using wavelet transforms [40], Mel-Frequency Cepstral Coefficients (MFCCs) [41,42], and Constant-Q Transform (CQT) analysis [43], which capture transient, perceptual, and frequency-localized aspects of the signal, respectively.

To assess underlying chaotic dynamics and recurrence properties, we extracted features based on Lyapunov Exponents [44], recurrence quantification analysis (RQA) [45], entropy metrics [46], and cycle-based statistics were incorporated to enhance discriminability and robustness. A dedicated set of pattern-based features was also included to represent the structural characteristics of the signals. These included Local Binary Patterns (LBP), Probabilistic Binary Patterns (PBP) [47,48], and texture-based descriptors such as contrast, correlation, homogeneity, and energy [48]. These features captured spatial and temporal regularities, symmetry, and variation in the time-frequency representations, providing discriminative power in distinguishing between classes. Additional features were extracted from the Bispectrum bounding box geometry (e.g., area, perimeter, aspect ratio) [48], spectral shape (e.g., spectral flux, centroid, bandwidth) [49], and signal dynamics (e.g., amplitude modulation, autocorrelation, CDF metrics) [50]. Features from the tunable Q-factor wavelet transform (TQWT) further captured oscillatory patterns across resolutions. A complete list of extracted features (including formulation and rationale) is provided in Appendix D, Table A1.

### 3.5. Automatic Feature Normalization

**Figure A2:**
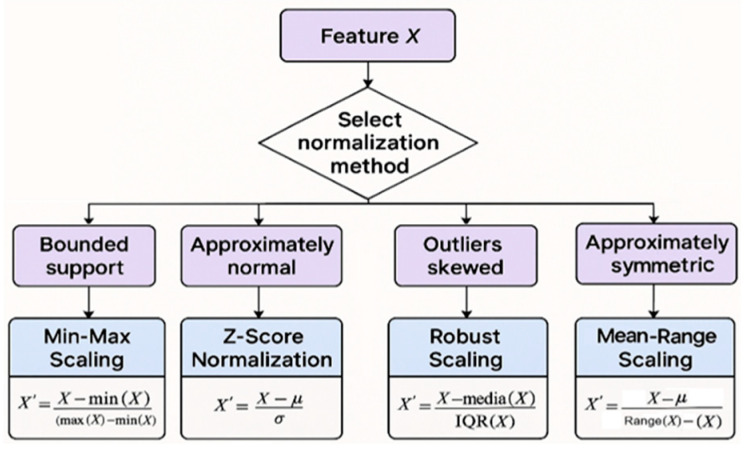
Flow chart of automatic feature normalization.

To ensure optimal preprocessing, an adaptive algorithm selected the most appropriate normalization method based on information shared between features and labels [51,52,53,54]. Details of the normalization methods evaluated, mutual information calculation, and flow chart are provided in Appendix B, Figure A2.

### 3.6. Selecting Best Features

**Figure A3:**
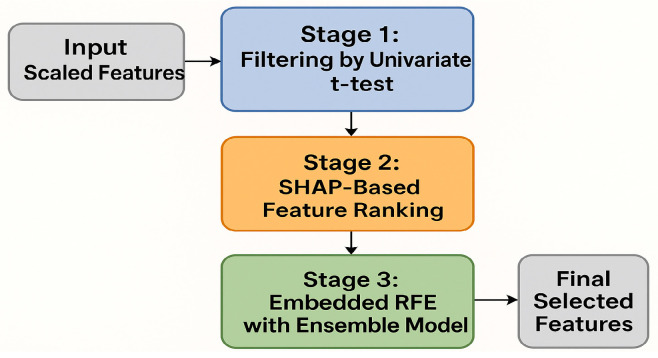
Flow chart of feature selection methodology.

**Figure A4:**
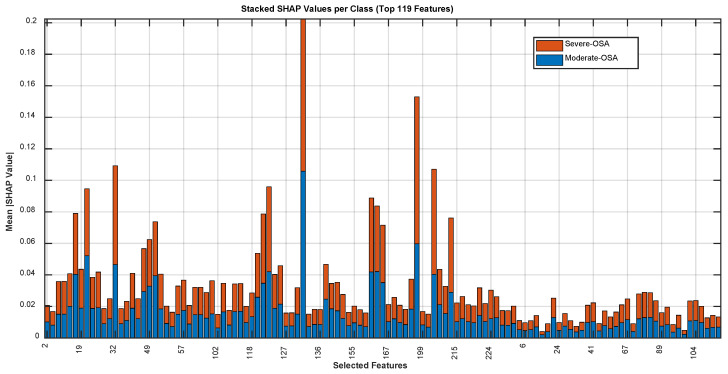
Shapley values calculated for selected features of base model 6.

A three-stage pipeline integrating filter (*t*-test), embedded (SHAP-based ranking), and wrapper (Recursive Feature Elimination with RUSBoost) methods was used to select informative and stable features [55,56,57,58,59,60,61]. The full description, algorithmic workflow (Figure A3), and associated figure (Figure A4) are included in Appendix C.

### 3.7. Experiments Models Training

The training of experiments models follows a systematic methodology that combines hyperparameter optimization, bootstrap aggregation (bagging), and ensemble-based validation to ensure robust model selection. This comprehensive approach is carefully designed to evaluate various classifiers under various configurations while addressing common issues such as overfitting, high variance, and unreliable performance estimates. By integrating multiple techniques into a structured pipeline, the methodology aims to produce models that generalize well to unseen data and provide reproducible, high-quality results. Six binary base experiments were trained to capture pairwise distinctions between different severity levels of OSA for the experiment’s models. These include the following:

1. Non-OSA vs. Mild-OSA;
2. Non-OSA vs. Moderate-OSA;
3. Non-OSA vs. Severe-OSA;
4. Mild-OSA vs. Moderate-OSA;
5. Mild-OSA vs. Severe-OSA;
6. Moderate-OSA vs. Severe-OSA.

The complete training methodology was applied to every pairwise comparison. This ensured that base classifiers were explicitly optimized for the discriminative characteristics relevant to each subset of the data. The process is divided into several key stages, as described below.

#### 3.7.1. Classifier Configuration and Hyperparameter Optimization

The first stage of base model training involves configuring a diverse set of classifiers and systematically optimizing their hyperparameters. A total of eighteen classifier configurations were explored to capture a broad spectrum of modeling paradigms [62]. These included the following:

- Traditional models: Decision Trees, Naïve Bayes, and Logistic Regression (L1 and L2 regularized).
- Distance- and projection-based models: k-Nearest Neighbors (kNN), Linear Discriminant Analysis (LDA), and Quadratic Discriminant Analysis (QDA).
- Margin-based models: Support Vector Machines (SVMs) with linear, radial basis function (RBF) and polynomial kernels.
- Ensemble methods: Random Forests, Bagged Trees, Gradient Boosting Machines (GBM), RUSBoost, and Subspace kNN.
- Neural networks: Both shallow and deep architectures.

The heterogeneous nature of breathing sound features and associated clinical data guided the selection of these classifiers. To address the varying degrees of complexity in the input space, the methodology balanced interpretable models (e.g., Logistic Regression, Decision Trees) with nonlinear learners (e.g., Neural Networks, SVMs, GBMs) [63,64]. Special emphasis was placed on ensemble methods, which are known for their robustness and ability to reduce overfitting, particularly in imbalanced and moderately sized datasets.

Each classifier was paired with a custom-defined hyperparameter search space tailored to its critical tuning variables (as detailed in Table 1). Using the Expected Improvement Plus (EI+) acquisition function, Bayesian optimization was employed to navigate these spaces. This method efficiently balances the exploration of high-dimensional parameter spaces with the exploitation of high-performing regions, leading to faster convergence than grid or random search methods [64]. The optimization objective was to minimize the misclassification rate under 5-fold stratified cross-validation, using the following loss function:

(2) $$L(\theta )=\frac{1}{K}\sum_{k=1}^{K}I(y_{i}\neq {\hat{y}}_{i}(\theta ))$$

where θ represents the hyperparameters, K is the number of cross-validation folds, $y_{i}$ is the actual label, and ${\hat{y}}_{i}(\theta )$ is the predicted label given the hyperparameters and I(⋅) is the indicator function [63,64]. Depending on classifier complexity, convergence was typically achieved with 50 iterations. This optimization method was selected because it efficiently balances exploration and exploitation in high-dimensional spaces, making it well-suited for complex models. Using 5-fold stratified cross-validation ensured that each model’s performance estimate was reliable and not biased by a particular subset of the data. By covering a diverse set of modeling strategies, the methodology maximizes the likelihood of identifying experimental models with complementary strengths, which is critical for the subsequent ensemble learning and modeling phases [62,63].

#### 3.7.2. Bootstrap Aggregation with OOB Validation

Following hyperparameter optimization, each classifier underwent bootstrap aggregation (bagging) to enhance generalization and reduce model variance [65]. For each base model, B = 50 bootstrap samples were generated by resampling the training data with replacement, where each sample $D_{b}$ had size N, equal to the number of original training instances. Using each $D_{b}$, a base learner $f_{b}$ was trained with its respective optimized hyperparameters $\theta^{*}$ [66]. For the b−th bootstrap sample $D_{b}$:

1. A resample $D_{b}$ was generated with replacement $|D_{b}|=N$, where N is the number of training instances.

2. Classifier $f_{b}$ was trained on $D_{b}$ using optimized $\theta^{*}$.

3. OOB samples $D_{\mathrm{oob}}^{\left(b\right)}=D\setminus D_{b}$ were retained for validation.

An essential advantage of this strategy is its natural support for out-of-bag (OOB) validation, which enables unbiased performance estimation without requiring a separate validation set. For instance, $x_{i}$ had an OOB prediction computed by aggregating predictions from all base learners for which $x_{i}\notin D_{b}$, i.e., from models that did not see that instance during training [67]. Formally, the OOB prediction is given by

(3) $${\hat{y}}_{i}^{\mathrm{OOB}}=\mathrm{mode}(\{f_{b}(x_{i})|x_{i}\in D_{\left(b\right)}^{\mathrm{oob}}\})$$

where $D_{\left(b\right)}^{\mathrm{oob}}=D\setminus D_{b}$ represents the set of OOB samples for the b-th bootstrap. This mechanism yields a robust estimate of each classifier’s generalization performance while fully utilizing the available training data. For each classifier configuration, the following OOB-based metrics were computed from the ensemble’s predictions:

- OOB Accuracy: Overall correct classification rate.
- OOB Sensitivity: True positive rate, capturing the ability to detect positive cases.
- OOB Specificity: True negative rate, reflecting the ability to identify negative cases correctly.

By averaging predictions across 50 model instances and leveraging OOB samples, this ensemble approach not only improves model stability and robustness but also provides reliable, data-efficient validation suitable for imbalanced or limited-size datasets [65,66,67,68].

#### 3.7.3. Class Imbalance Mitigation

For datasets with skewed class distributions [69,70]:

1. Cost-sensitive learning: Class-weighted loss functions scale misclassification costs inversely to class frequencies [69].
2. Stratified bootstrapping: Maintains original class ratios in bootstrap samples [69].
3. OOB-balanced metrics: Performance evaluation weights classes by inverse frequency [69].

This three-pronged approach prevents bias toward majority classes while preserving detection capability for rare categories [69,70].

#### 3.7.4. Robust Model Selection via Repeated Trials

Recognizing that machine learning training processes are inherently stochastic due to factors like data shuffling, bootstrap sampling, and optimization randomness, the methodology incorporated multiple independent training trials [71,72]. For each classifier configuration, five independent trials were conducted. Each trial involved reinitializing Bayesian hyperparameter optimization to ensure that different regions of the search space could be explored, thus avoiding convergence to suboptimal local minima [63]. Additionally, 50 new bootstrap models were generated in each trial to introduce variability into the ensemble learning process. After training, performance metrics were evaluated using OOB samples and a held-out test set. Among the five trials, the one achieving the highest OOB accuracy was selected for final model comparison. This process ensured that the best-performing model was chosen based on generalizable results rather than random fluctuations in performance.

#### 3.7.5. Final Model Selection

For each pairwise model of the six models, the classifier with the highest OOB accuracy was selected as the final classifier. This selection criterion favors models that generalize well during training without overfitting, a benefit inherent to bootstrap aggregation and OOB validation [73]. Additionally, by selecting the best classifier for each experiment based on OOB performance rather than test performance, the methodology avoids the risk of overfitting the test set, preserving its integrity for unbiased final evaluation [74]. All results, including detailed accuracy, sensitivity, and specificity metrics, were compiled into a unified table to facilitate cross-dataset comparisons and meta-analyses. This organized approach enables robust insights into the relative performance of different models across varying datasets and conditions.

### 3.8. Model Evaluation

To assess the effectiveness of the models, the evaluation framework relies on standard classification performance metrics. This approach emphasizes both predictive accuracy and the balanced assessment of model performance across different classes, particularly in the presence of class imbalance.

#### 3.8.1. Evaluation Protocol

(a) Out-of-Bag (OOB) Validation

During ensemble training, each base learner was evaluated on the subset of training samples excluded from its bootstrap resample. These out-of-bag predictions were used to estimate performance without requiring a separate validation set. The resulting OOB metrics provide an unbiased estimate of generalization error.

(b) Independent Test Evaluation

After model training, performance was assessed using the same metrics on a held-out test set. To account for randomness in training (e.g., bootstrap sampling or stochastic optimization), the evaluation was repeated 25 times over independent trials with different random seeds.

#### 3.8.2. Performance Metrics

Models were evaluated using three core metrics—accuracy, sensitivity, and specificity—on both out-of-bag (OOB) samples and independent test sets [75].

## 4. Results

The performance of the proposed OSA severity screening framework was evaluated across multiple classification tasks using both tracheal breathing sounds (TBS) and anthropometric features. Results are reported for six pairwise comparisons of OSA severity levels (Non-OSA, Mild, Moderate, Severe), emphasizing classifier robustness, generalization, and clinical relevance. Model evaluation was conducted using out-of-bag (OOB) validation during training and a fully independent blind test set to assess real-world applicability. Performance metrics, including accuracy, sensitivity, and specificity, were computed to reflect diagnostic balance. The results of the experiment’s models support the utility of detecting OSA severity during wakefulness using these models, providing a rapid, non-invasive method. The following sub-sections show the results of the proposed methodology.

### 4.1. Feature Selection Results

The feature selection process identified distinct and informative sets of features for each model, capturing the essential aspects of breathing sounds during the mouth and nose inspiration and expiration phases. Table A2 provides a summary of selected features for each base model.

The best-performing model (Model 1) leveraged a combination of spectral and image-based features to effectively classify breathing patterns. Key features included spectral entropy and crest values extracted from specific frequency bands during mouth expiration gaps, wavelet-based spectral bandwidth, and kurtosis metrics derived from nose inspiration and expiration phases. Image-derived features, such as the number of holes, bounding box area, and texture contrast across the mouth and nose regions further enhanced the model’s discriminatory capability. Additional contributing features included fractal dimension estimates, peak frequency, and statistical measures derived from Mel-Frequency Cepstral Coefficients (MFCCs). A detailed list of selected features for all models (Models 1–6) is provided in Appendix D, Table A2.

The feature selection process across all six models identified a diverse set of acoustic, spectral, fractal, and image-based characteristics that effectively capture the nuances of breathing sounds. Most of these features were extracted predominantly from the mouth inspiration segments, which provided rich spectral and fractal information, while some were derived from expiration and combined inspiration–expiration phases. The selected features include spectral measures such as spectral centroid, entropy, skewness, flux, power spectral density statistics, fractal dimensions, and wavelet-based coefficients. Statistical descriptors of MFCCs, zero crossing rates, Bispectrum entropy, and harmonic–percussive source separation features further enrich the dataset. Additionally, morphological features extracted from image representations of the respiratory signals, such as bounding box area, number of holes, connected components, and Euler numbers, contributed to capturing structural variations in the signal. Together, these features comprehensively represent both the temporal and frequency-domain properties of the respiratory signals, enabling robust discrimination between subject classes.

These comprehensive acoustic and morphological features were combined with seven key anthropometric variables: body mass index (BMI), age, sex, smoke history, neck circumference (NC), and Mallampati score (MPS). Integrating these physiological and demographic factors with the rich features of breathing sounds enhances the models’ ability to reflect intrinsic body characteristics and breathing dynamics. This fusion yields 41 features for each model, resulting in a robust, multidimensional dataset for subsequent classification and analysis tasks.

### 4.2. Experiments Models Results

#### 4.2.1. Training and Testing Results

The selected base classifiers, Random Forest, Support Vector Machines (SVMs) with polynomial kernels of degrees 3, 5, and 7, Subspace K-Nearest Neighbors (Subspace KNN), and Linear Discriminant Analysis (LDA), demonstrate consistently strong performance according to their out-of-bag (OOB) estimates. These classifiers were chosen for their reliability and robustness across different datasets. The OOB accuracies remain high across all models, typically exceeding 80%, indicating that the classifiers are well-calibrated and effectively generalized during internal validation. Additionally, the OOB sensitivity and specificity values are balanced, suggesting that these models strike a good balance between identifying true positives and minimizing false positives. Table 5 presents the out-of-bag (OOB) performance metrics for these classifiers.

Then, the test results corresponded to the models that achieved high OOB performance across each dataset. These independent evaluations further validate the generalization capability of the selected classifiers. The test accuracies, sensitivities, and specificities closely mirror the trends observed in the OOB evaluations, with deviations generally remaining within 10%, an acceptable range in practical classification tasks. Several models, including Random Forest and SVMs with polynomial kernels, achieved perfect sensitivity or specificity on specific datasets, highlighting their potential for robust classification in real-world applications. Table 6 shows the test performance metrics for these classifiers.

**Figure 6:**
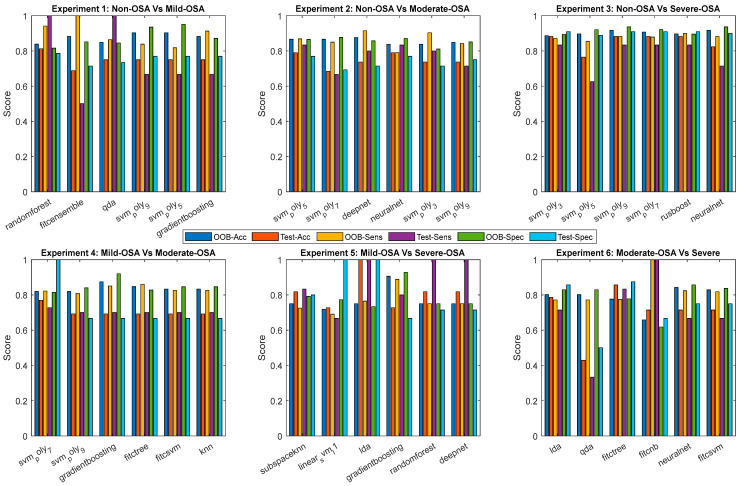
Expanded classifier performance metrics across six binary experiments. For each experiment, the top six classifiers were selected based on their mean performance. Grouped bar plots display both out-of-bag (OOB) and test set results for accuracy, sensitivity, and specificity. This visualization highlights the differences between training (out-of-bag, OOB) and generalization (test) performance.

**Figure 7:**
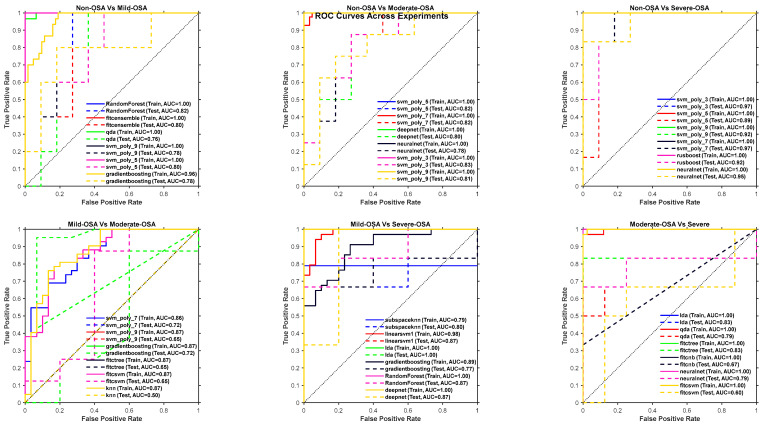
Receiver Operating Characteristic (ROC) curves of the top six classifiers for representative binary experiments. The curves illustrate the discrimination ability of each classifier across sensitivity–specificity trade-offs, complementing Table 4 and Table 5 by providing a visual comparison of performance beyond single-value metrics.

To provide a comprehensive overview, we additionally report performances from all evaluated classifiers in Figure 6 and Figure 7. These figures illustrate the distribution of accuracy, sensitivity, and specificity across classifiers, complementing the summary in Table 5 and Table 6.

#### 4.2.2. K-Fold Results

The selected base classifiers demonstrated consistently strong performance across 3-fold cross-validation. The OOB accuracies remained high, generally exceeding 80%, indicating effective generalization and stability across folds. Sensitivity and specificity values were also well-balanced, suggesting that the models achieved a good trade-off between detecting true positives and minimizing false positives. Table 7 presents the 3-fold OOB performance metrics, which are consistent with the trends observed in the previous training–testing evaluations.

The test results correspond to models that achieved strong performance during internal validation. These independent evaluations further confirm the generalization capability of the selected models. The test accuracies, sensitivities, and specificities closely reflected the patterns observed in cross-validation, with deviations generally remaining within 10%, an acceptable range for practical classification tasks. Table 8 presents the test performance metrics, which are consistent with the trends observed in previous evaluations.

## 5. Discussion

The proposed OSA severity screening during wakefulness framework demonstrates a promising advance in non-invasive, wakefulness-based diagnostic tools, particularly by integrating tracheal breathing sound analysis and anthropometric data. The structured evaluation across six pairwise OSA severity classifications offers robust performance metrics and insights into the physiological and acoustic distinctions among severity levels. This discussion synthesizes these findings, emphasizing the methodology’s strengths and implications for clinical practice.

Incorporating SHAP into the feature selection pipeline provided an interpretable and data-driven mechanism to quantify each feature’s contribution toward OSA severity classification. Unlike traditional ranking approaches, SHAP integrates cooperative game theory principles to assign fair importance values to features based on their marginal contributions across multiple model predictions. This allowed us to confirm that physiologically relevant features, such as spectral entropy, bispectrum-derived texture measures, and anthropometric variables like BMI and neck circumference were consistently influential across models. Significantly, SHAP enhanced transparency by linking acoustic and morphological variations in tracheal breathing sounds with interpretable physiological correlations, thereby bridging the gap between clinical understanding and algorithmic decision-making. When combined with Recursive Feature Elimination (RFE), the SHAP-guided ranking ensured that only the most stable and clinically meaningful features were retained, which improved model robustness while reducing dimensionality. This integration strengthens the interpretability and reliability of the proposed wakefulness-based OSA severity screening framework.

The feature selection process revealed diverse spectral, temporal, fractal, and morphological characteristics extracted from mouth- and nose-breathing segments during different respiratory phases. Notably, features such as spectral entropy, crest, kurtosis, fractal dimension, and MFCC-based statistics emerged repeatedly across models. These features are physiologically meaningful, as they capture underlying variations in airway obstruction, turbulence, and breathing effort associated with different severities of OSA. Notably, morphological features derived from bispectrum image representations of the respiratory signals, such as bounding box area, Euler number, and connected components, provide a novel dimension to acoustic analysis. These image-based descriptors translate subtle acoustic changes into quantifiable structural patterns, enhancing interpretability and model transparency. Furthermore, the consistent contribution of mouth inspiration segments as primary sources of discriminative features underscores their diagnostic richness, likely due to greater variability in the upper-airway resistance during inspiration in OSA patients. This is because mouth-breathing bypasses the nasal passages and exposes the more collapsible pharyngeal airway to direct airflow. During inspiration, this region is more prone to dynamic narrowing and turbulence in individuals with OSA, resulting in acoustic patterns that better reflect underlying structural abnormalities compared to nasal breathing. This aligns with the prior literature highlighting mouth-breathing as a compensatory mechanism in individuals with compromised nasal airflow and may reflect airway collapse dynamics during wakeful states [19,20].

The selected features encompass a broad range of physiological representations of breathing sound dynamics, each reflecting essential aspects of upper-airway structure and function that are affected by obstructive sleep apnea (OSA). Spectral features, such as spectral entropy, skewness, kurtosis, crest, centroid, and bandwidth, quantify the distribution and organization of energy across frequencies. In patients with OSA, upper-airway obstruction during inspiration and expiration leads to increased turbulence, reflected in broader spectral distributions (higher entropy), asymmetric power distribution (skewness), and heavier spectral tails (kurtosis). These features can capture abnormal airflow patterns due to pharyngeal collapse, especially during inspiration, which is more sensitive to airway resistance [20].

Fractal dimensions and nonlinear dynamics measures such as the Hurst exponent, Lyapunov exponent, and Katz fractal dimension reflect the irregularity and complexity of breathing signals, which tend to increase with the severity of OSA due to variable airflow and compensatory muscle activity [20,21,76]. MFCCs (Mel-Frequency Cepstral Coefficients), though commonly used in speech processing, effectively capture spectral envelope variations that correlate with airway resonance characteristics, which are particularly altered in OSA due to anatomical and functional airway changes [77]. Bispectrum features and bicoherence quantify quadratic phase coupling between frequencies, providing insights into the nonlinear and harmonic interactions typical of turbulent breathing in OSA.

Time-domain features such as zero-crossing rate, peak frequency, and signal energy characterize oscillatory behavior and airflow strength [19]. These are sensitive to inspiratory effort and upper-airway resistance [19]. Additionally, wavelet-based features offer a multi-resolution analysis of signal transients, making them suitable for identifying events such as partial obstructions or arousals during respiration. CQT (Constant-Q Transform) and entropy-based features derived from wavelet or CQT domains reflect subtle changes in airflow rhythm and complexity [78], which may not be detectable in standard spectral measures.

Morphological and image-based features such as bounding box area, number of holes, Euler number, and texture measures are extracted from spectrogram or bispectrum image representations and serve as indirect quantifiers of structural variation in acoustic patterns. These are physiologically linked to airway geometry and dynamic obstruction events [20,79], as turbulent flow often generates unique spatial textures in time–frequency representations. Finally, anthropometric features such as BMI, neck circumference, Mallampati score, and age are directly related to anatomical risk factors for OSA, including fat deposition around the neck, airway collapsibility, and tongue size.

Together, these features form a multidimensional physiological signature of breathing under different severities of OSA. Their combined use enhances the ability to non-invasively and objectively screen OSA severity during wakefulness with high reliability. While anthropometric data improved classification accuracy, reliance on such features may limit usability in home-based screenings. A direct comparison with tools like STOP-Bang and Berlin questionnaires was not conducted in this study; future work should benchmark performance against these established screening methods to better contextualize the advantages.

For the training testing results, the base classifiers demonstrated strong internal validation performance through out-of-bag (OOB) evaluation, with accuracies generally exceeding 80%, and a balanced sensitivity and specificity. These results indicate that the models are well-calibrated and generalize effectively within the training data. The SVM with polynomial kernels (particularly degrees 3 and 5) and Random Forests emerged as consistently high performers, achieving high accuracy and balanced diagnostic metrics across severity comparisons. Such consistency highlights their ability to model nonlinear interactions and feature dependencies in complex acoustic-physiological data. External validation using a blind test set reinforced the models’ real-world applicability. The test performance observed mirrored OOB results, with deviations typically within 10%. For instance, Model 1 (Random Forest) achieved 100% sensitivity, indicating its ability to accurately capture actual OSA-positive cases, which is crucial for clinical screening scenarios. Similarly, Model 4 (SVM Poly 7) achieved 100% specificity, emphasizing its strength in confidently identifying non-OSA subjects. These extreme yet balanced outcomes across classifiers suggest complementary strengths ripe for ensemble integration.

The 3-fold cross-validation further confirmed the robust performance of the base classifiers, with accuracies generally remaining above 80%, and sensitivities and specificities showing balanced values across folds. These results indicate that the models are stable under repeated resampling and generalize effectively within the training data. Notably, SVMs with polynomial kernels (particularly degrees 3 and 9) and Random Forests consistently achieved high accuracy and well-balanced diagnostic metrics, reflecting their ability to capture nonlinear relationships and complex interactions in the data. External validation using the test sets reinforced these findings, with deviations from OOB results typically within 10%. For example, Model 2 (Random Forest) achieved 87% sensitivity, demonstrating reliable detection of true positives, while Model 3 (Gradient Boosting) reached 93% specificity, emphasizing the accurate identification of true negatives. This complementary performance across classifiers underscores their potential value in ensemble modeling for enhanced predictive reliability.

An essential next step is to investigate whether predicted OSA severity correlates with perioperative complication rates, which would reinforce the translational value of this method for surgical risk stratification.

## 6. Limitations

Although the system shows strong potential, several limitations must be acknowledged. First, all data in this study were collected under controlled conditions at a single center using one microphone type (Sony ECM77B). This may limit generalizability, as real-world environments, such as different clinics or home settings, introduce variability in background noise, microphone type, and user behavior. Future work should therefore validate the framework across centers, devices, and include analyses of self-placement errors to assess robustness in more realistic scenarios.

Another issue is the reliance on consistent microphone placement over the trachea. While this was carefully managed during data collection, it is possible that in real-world use, especially in self-administered or remote settings, the placement may not always be accurate. Small changes in position could affect the sound quality and, in turn, the model’s predictions. Future versions of the system should account for this, possibly by incorporating signal quality checks or providing user guidance. There is also the matter of anthropometric data. Some features, like neck circumference or jaw position, might not always be easy to measure, particularly outside of a clinical environment. Although combining these features with acoustic data improves accuracy, it could limit the tool’s practicality in settings where full measurements are not available. Exploring ways to work with partial data or identifying simpler substitutes would make the system more accessible. Although the dataset is relatively small and imbalanced across severity groups, we mitigated overfitting through 3-fold stratified cross-validation during hyperparameter tuning, bootstrap aggregation with OOB validation, and repeated independent training trials. Nevertheless, larger and multi-center datasets are needed for stronger statistical power.

Finally, while the tool performs well as a screening aid, it is not a replacement for clinical diagnosis. Its role should be clearly defined within the broader diagnostic process, helping to flag potential cases but not making final decisions. More work is needed to understand how clinicians would use the results in practice and how to communicate the model’s output in a way that is both useful and trustworthy.

## 7. Conclusions

This study demonstrates the foundation for the effectiveness of a fast, objective, and non-invasive method for screening obstructive sleep apnea (OSA) severity using tracheal breathing sounds (TBS) recorded during wakefulness. By combining features from binary severity comparisons with anthropometric data, the proposed experiment’s model approach achieved high classification performance across multiple machine learning models. Notably, the SVM with a third-degree polynomial kernel delivered strong out-of-bag and test results, while Random Forests achieved perfect test sensitivity. These findings support the potential of TBS-based analysis as a practical and accessible alternative to polysomnography, enabling a reliable assessment of OSA severity in under 10 min and representing a significant advancement for early detection and perioperative risk management.

The developed framework successfully harnesses the multidimensional richness of breathing sound analysis and anthropometric data to screen for OSA severity levels with high accuracy and clinical relevance. The effective combination of feature selection, robust base classifiers, and a well-structured approach illustrates a scalable and interpretable method for non-invasive respiratory assessment. These findings represent a crucial step toward AI-driven, accessible sleep disorder diagnostics that can bridge the current gaps in OSA identification and management.

## Figures and Tables

**Table 1:** Participants’ severity groups and anthropometric information.

Group	Num. of Subjects	AHI	AGE	Sex	BMI	MPS	NC
Non-OSA	74	1.2 ± 1.3	46.8 ± 12.9	29 M, 45 F	30.6 ± 6.2	41 (1), 19 (2), 6 (3), 8 (4)	38.8 ± 4.0
Mild	35	8.7 ± 2.6	52.3 ± 11.6	21 M, 14 F	34.3 ± 8.4	18 (1), 6 (2), 9 (3), 1 (4)	42.1 ± 6.5
Moderate	50	21.5 ± 4.2	54.7 ± 11.3	36 M, 14 F	33.8 ± 6.4	17 (1), 17 (2), 8 (3), 8 (4)	43.1 ± 3.4
Severe	40	69.5 ± 33.3	48.9 ± 11.1	30 M, 10 F	39.7 ± 8.7	5 (1), 13 (2), 14 (3), 8 (4)	45.3 ± 3.6

AHI: apnea–hypopnea index; BMI: body mass index; NC: neck circumference; MPS: Mallampati score; M/F: male/female.

**Table 2:** Participants’ severity groups and anthropometric information for training set.

Group	Num. of Subjects	AHI	AGE	Sex	BMI	MPS	NC
Non-OSA	63	1.2 ± 1.2	46.8 ± 13.5	24 M, 39 F	30.6 ± 6.6	36 (1), 17 (2), 5 (3), 5 (4)	39.0 ± 3.5
Mild	30	8.8 ± 2.6	50.9 ± 10.4	17 M, 13 F	34.1 ± 8.2	14 (1), 7 (2), 8 (3), 0 (4)	41.5 ± 7.0
Moderate	42	22.0 ± 4.3	53.7 ± 10.3	31 M, 11 F	33.7 ± 6.8	11 (1), 15 (2), 8 (3), 8 (4)	433 ± 3.3
Severe	34	68.5 ± 32.5	50.9 ± 11.1	24 M, 10 F	39.9 ± 9.3	4 (1), 11 (2), 13 (3), 6 (4)	45. ± 3.4

AHI: apnea–hypopnea index; BMI: body mass index; NC: neck circumference; MPS: Mallampati score; M/F: male/female.

**Table 3:** Participants’ severity groups and anthropometric information for the testing set.

Group	Num. of Subjects	AHI	AGE	Sex	BMI	MPS	NC
Non-OSA	11	0.9 ± 1.0	45.4 ± 10.7	6 M, 5 F	30.6 ± 3.8	6 (1), 3 (2), 1 (3), 1 (4)	40.1 ± 4.5
Mild	5	6.7 ± 1.7	52.4 ± 12.6	3 M, 2 F	30.3 ± 10.2	3 (1), 1 (2), 1 (3), 0 (4)	41.9 ± 1.5
Moderate	8	20.7 ± 4.5	58.5 ± 7.3	4 M, 4 F	33.7 ± 6.2	4 (1), 3 (2), 1 (3), 0 (4)	41.5 ± 4.0
Severe	6	80.0 ± 35.1	46.4 ± 11.3	4 M, 2 F	44.4 ± 6.2	1 (1), 2 (2), 1 (3), 2 (4)	44.0 ± 3.7

AHI: apnea–hypopnea index; BMI: body mass index; NC: neck circumference; MPS: Mallampati score; M/F: male/female.

**Table 4:** Participants’ severity groups and anthropometric information for K-folds.

Group	Folds	Num. of Subjects	AHI	AGE	Sex	BMI	MPS	NC
Non-OSA	1	23	0.6 ± 0.8	44.9 ± 12.1	10 M, 13 F	29.2 ± 4.7	12 (1), 7 (2), 1 (3), 3 (4)	38.0 ± 4.7
	2	27	1.1 ± 1.3	45.7 ± 12.1	10 M, 17 F	32.3 ± 7.6	12 (1), 9 (2), 4 (3), 2 (4)	39.2 ± 4.3
	3	24	1.8 ± 1.3	50.0 ± 14.3	9 M, 15 F	30.0 ± 5.8	17 (1), 3 (2), 1 (3), 3 (4)	39.0 ± 2.9
Mild	1	16	8.7 ± 2.4	50.9 ± 12.5	10 M, 6 F	36.6 ± 9.9	7 (1), 1 (2), 7 (3), 1 (4)	43.5 ± 5.3
	2	10	8.6 ± 2.2	51.3 ± 11.6	5 M, 5 F	31.8 ± 8.4	6 (1), 1 (2), 2 (3), 1 (4)	38.5 ± 8.8
	3	9	8.8 ± 3.5	56.0 ± 10.3	6 M, 3 F	33.0 ± 4.2	5 (1), 4 (2)	43.4 ± 2.7
Moderate	1	16	19.9 ± 2.9	56.3 ± 10.8	10 M, 6 F	34.6 ± 7.8	4 (1), 6 (2), 2 (3), 4 (4)	42.3 ± 4.0
	2	18	22.8 ± 4.5	53.6 ± 9.7	13 M, 5 F	31.8 ± 5.7	8 (1), 5 (2), 3 (3), 2 (4)	42.7 ± 3.8
	3	16	21.6 ± 4.7	54.5 ± 13.9	13 M, 3 F	35.2 ± 5.2	5 (1), 6 (2), 3 (3), 2 (4)	43.6 ± 2.8
Severe	1	14	72.9 ± 35.0	45.5 ± 10.5	11 M, 3 F	39.1 ± 10.1	2 (1), 3 (2), 5 (3), 4 (4)	44.3 ± 4.2
	2	16	66.6 ± 29.6	50.2 ± 11.0	13 M, 3 F	40.1 ± 8.5	1 (1), 6 (2), 5 (3), 4 (4)	46.6 ± 3.2
	3	10	69.6 ± 39.1	51.9 ± 12.2	6 M, 4 F	40.2 ± 7.5	2 (1), 4 (2), 4 (3)	43.8 ± 3.5

AHI: apnea–hypopnea index; BMI: body mass index; NC: neck circumference; MPS: Mallampati score; M/F: male/female.

**Table 5:** OOB results for experimental models.

Experiment	Classifier	OOB Accuracy	OOB Sensitivity	OOB Specificity
1	Random Forests	83.9	94.1	81.6
2	SVM Poly 5	86.7	86.8	86.6
3	SVM Poly 3	88.7	87.1	89.4
4	SVM Poly 7	81.9	82.2	81.5
5	Subspace KNN	75.0	72.5	79.2
6	LDA	80.3	77.1	82.9

**Table 6:** Test results for experimental models.

Experiment	Classifier	Test Accuracy	Test Sensitivity	Test Specificity
1	Random Forests	81.2	100	78.6
2	SVM Poly 5	78.9	83.3	76.9
3	SVM Poly 3	88.2	83.3	90.9
4	SVM Poly 7	76.9	72.7	100
5	Subspace KNN	81.8	83.3	80.0
6	LDA	78.6	71.4	85.7

**Table 7:** OOB 3-fold cross-validation results of experimental models.

Experiment	Fold	Classifier	OOB Accuracy	OOB Sensitivity	OOB Specificity
1	1	Logistic	85.7	79.9	94.0
	2	Decision Trees	86.1	79.7	95.7
	3	Deep Neural Net	85.5	72.8	94.0
2	1	Random Forest	84.7	92.2	73.5
	2	SVM RBF	84.8	89.4	78.1
	3	Shallow Neural Net	88.1	92.0	82.4
3	1	SVM Poly 9	83.1	77.1	92.2
	2	SVM Poly 3	98.6	97.9	100.0
	3	Gradient Boosting	93.8	94.0	93.3
4	1	Ensemble Model	81.1	88.2	70.5
	2	RUSBoost	91.2	88.0	93.8
	3	Logistic	83.3	76.9	88.2
5	1	Decision Trees	80.0	78.9	80.8
	2	Deep Neural Net	85.7	88.0	83.3
	3	Random Forest	82.1	84.6	80.0
6	1	SVM RBF	78.3	82.4	73.1
	2	Shallow Neural Net	81.1	83.8	77.1
	3	SVM Poly 9	82.8	85.3	80.0

**Table 8:** Test 3-fold cross-validation results of experimental models.

Experiment	Fold	Classifier	Test Accuracy	Test Sensitivity	Test Specificity
1	1	Logistic	82.1	84.6	78.3
	2	Decision Trees	77.1	77.0	77.2
	3	Deep Neural Net	77.8	79.6	75.0
2	1	Random Forest	82.1	87.0	75.0
	2	SVM RBF	77.8	81.5	72.2
	3	Shallow Neural Net	84.6	88.2	79.2
3	1	SVM Poly 9	81.9	85.7	79.4
	2	SVM Poly 3	72.1	70.4	75.0
	3	Gradient Boosting	73.5	75.0	70.0
4	1	Ensemble Model	76.4	78.6	75.0
	2	RUSBoost	87.4	81.8	91.2
	3	Logistic	73.5	70.7	77.8
5	1	Decision Trees	76.7	75.1	79.0
	2	Deep Neural Net	84.6	90.0	81.3
	3	Random Forest	73.7	71.2	71.2
6	1	SVM RBF	72.7	71.4	74.7
	2	Shallow Neural Net	73.5	74.4	72.2
	3	SVM Poly 9	71.9	72.2	71.3

## Data Availability

To access the anonymized data for research purposes, one may contact the PI of the study (last author).

## Abbreviations

The following abbreviations are used in this manuscript:

AHI	Apnea–Hypopnea Index
BMI	Body Mass Index
CQT	Constant-Q Transform
DFA	Detrended Fluctuation Analysis
ECOC	Error-Correcting Output Codes
GBM	Gradient Boosting Machine
kNN	k-Nearest Neighbors
LASSO	Least Absolute Shrinkage and Selection Operator
LDA	Linear Discriminant Analysis
LR	Logistic Regression
MFCC	Mel-Frequency Cepstral Coefficients
MPS	Mallampati Score
NC	Neck Circumference
OOB	Out-of-Bag
OSA	Obstructive Sleep Apnea
PSG	Polysomnography
RF	Random Forest
RFE	Recursive Feature Elimination
RQA	Recurrence Quantification Analysis
SHAP	SHapley Additive exPlanations
SVM	Support Vector Machine
TBS	Tracheal Breathing Sounds
TQWT	Tunable Q-Factor Wavelet Transform
VMD	Variational Mode Decomposition

## Appendix A. Anthropometric Missing Value Imputation

The imputation of missing anthropometric data was achieved within each severity group (Non-, Mild, Moderate and Severe OSA) using a k-nearest neighbors (k-NN) based approach [30]. The k-NN imputation estimates missing entries by leveraging the similarity among samples within the same OSA category, thus preserving the inherent structure and distribution of the data. This method enhances data completeness while minimizing the potential bias introduced by missingness [30].

By combining severity-specific grouping with localized imputation, the preprocessing approach ensures robust and consistent feature sets that improve the reliability of downstream modeling and statistical analyses [30,31]. Figure A1 illustrates the steps involved in filling in missing arthrometric data.

## Appendix B. Automatic Feature Normalization

An automatic selection algorithm was implemented to choose the most suitable normalization method for a given dataset, thereby optimizing feature preprocessing. For each feature $X_{i}\in X\subset R^{n\times d},$ four normalization techniques were evaluated: min–max scaling, z-score normalization, mean-range scaling, and robust scaling. Each feature was normalized individually and discretized into ten bins, and its dependency on the categorical labels y was quantified using mutual information [51,52,53]:

(A1) $$I\left(X;Y\right)=\underset{i,j}{\sum }p\left(x_{i},y_{j}\right)\log\left(\frac{p\left(x_{i},y_{j}\right)}{p\left(x_{i}\right)p\left(y_{j}\right)}\right)$$

The normalization method that yielded the highest cumulative mutual information across all features was selected. This adaptive selection ensured that feature–label relationships were preserved and enhanced during preprocessing [51]. Then, the chosen normalization was performed using one of four methods:

- Min–Max Scaling: Rescales data to the [0, 1] range [54] using

(A2) $$x^{\prime }=\frac{x-\min\left(x\right)}{\max\left(x\right)-\min\left(x\right)}$$

- Z-Score Normalization: Standardizes features to a zero mean and unit variance [54]:

(A3)
$$x^{\prime }=\frac{x-\mu }{\sigma }$$

- Mean-Range Scaling: Centers by the mean and scales by the range [54]:

(A4)
$$x^{\prime }=\frac{x-\mu }{\mathrm{range}(x)}$$

- Robust Scaling: Centers by the median and scales by the interquartile range (IQR) [54]:

(A5)
$$x^{\prime }=\frac{x-\mathrm{median}(x)}{\mathrm{IQR}(x)}$$

For each method, parameters (mean, standard deviation, minimum, maximum, median, IQR) were computed from the training data if not pre-specified, enabling their consistent application to the testing sets. The proposed automatic normalization selection ensures the preprocessing step is systematically adapted to the underlying data distribution. The method enhances feature relevance and model discriminability by maximizing the mutual information between rescaled features and categorical labels [51]. Furthermore, it increases resilience to outliers, non-Gaussian distributions, and varying feature ranges. This adaptive strategy enhances the robustness and generalization capabilities of subsequent learning models. Figure A2 shows a flow chart of the automatic feature normalization logic.

## Appendix C. Selecting Best Features

We implemented a robust, three-stage feature selection pipeline to ensure that the learning algorithms, particularly ensemble models, operated on a compact and informative set of inputs. This section describes the methodology in detail and explains the rationale for its use in models’ learning. The feature selection process combines filter, wrapper, and embedded methods to progressively refine the feature set. A pipeline flow chart is shown in Figure A3.

The pipeline consisted of three main stages: univariate filtering using *t*-test, model-based feature ranking via SHAP values, and Recursive Feature Elimination (RFE) with RUSBoost ensemble. This hierarchical approach helped eliminate redundant, noisy, or non-informative features, thereby enhancing model generalizability and interpretability.

### Appendix C.1. Stage 1: Filtering by Univariate t-Test

We applied a two-sample unpaired *t*-test to each feature in the first stage. The objective was to detect statistically significant differences in feature values between the two label groups. Features with a *p*-value ≤ 0.05 were retained for the next stage [55,56]. This method is computationally efficient and effective for removing features that are unlikely to contribute to class separation. However, it does not consider interactions between features or their impact on the learning algorithm’s performance [55,56].

### Appendix C.2. Stage 2: SHAP-Based Feature Ranking

Features retained from the *t*-test were further ranked using SHAP (SHapley Additive exPlanations) values. A classifier was trained using an Error-Correcting Output Codes (ECOC) ensemble model, and SHAP values were computed over multiple iterations [57,80]. In each iteration:

- The classifier was trained and evaluated.
- SHAP values were computed for each feature.
- The top N/2 features with the highest mean absolute SHAP value were stored.

Finally, we selected the features that appeared most frequently across iterations, ensuring robustness to sampling variance. This step introduces model explainability into the selection process, allowing for interpretability and greater confidence in the selected features [57,80]. Figure A4 shows the calculated shapely values for selected features when using the proposed method for base model 6.

### Appendix C.3. Stage 3: Embedded Recursive Feature Elimination (RFE) with Ensemble Model

The final stage employed Recursive Feature Elimination (RFE) using a RUSBoost ensemble classifier [58,59]. This method was embedded within the model training process, recursively ranking and removing the least essential features until the desired number of features was retained. RFE was conducted within a cross-validation loop to enhance generalizability and avoid overfitting a specific training partition. The most consistently retained features across folds were selected. This embedded method leverages the inherent feature importance estimates of the RUSBoost model and tailors feature selection to the classification task [58,59].

The proposed multi-stage feature selection pipeline offers several advantages, particularly within ensemble learning frameworks such as stacked generalization. The pipeline effectively balances speed, robustness, and task relevance by integrating filter methods such as *t*-tests, wrapper methods like RFE, and embedded techniques including SHAP-based ranking. Early-stage filtering removes non-discriminative features, reducing noise and enhancing the efficiency of subsequent modeling stages. SHAP values support model transparency and interpretability, which are essential in biomedical and clinical AI applications. Moreover, RFE aligns the selected features with the learning algorithm, ensuring they are optimized for predictive performance. As a result, the selected features are compact and stable and can be applied to the different experimental models used within the ensemble. In stacked ensemble systems, where individual experimental models are trained independently and a meta-learner combines their predictions, the quality of the features provided to each base model plays a critical role [60]. If experimental models are trained on noisy or inconsistent features, the ensemble’s performance can suffer due to weak or conflicting individual predictions. The proposed pipeline addresses this by ensuring each base model receives a consistent and informative feature set. Consequently, the meta-learner benefits from high-quality base predictions, which improves the overall strength and stability of the ensemble. This also reduces the generalization gap across both training and unseen data distributions. Therefore, the proposed feature selection pipeline is crucial for enhancing the accuracy, interpretability, and robustness of ensemble learning systems [61].

## Appendix D

**Table A1:** Summary of extracted features for all models.

Power Spectrum Features	Bi-Spectrum Features	Wavelet	Wavelet	MFCC	TP	CDF
MeanPower	Hf2 2	Wavelet Approx Mean L1	Wavelet Approx Mean L4	MFCC Mean C1	TP Histogram 1	CDFMean
StandardDeviation	En F Bi D 3	Wavelet Approx Std L1	Wavelet Approx Std L4	MFCC Std C1	TP Histogram 2	CDFStd
Maximum	Mean Bi D F 3	Wavelet Approx Skewness L1	Wavelet Approx Skewness L4	MFCC Skewness C1	TP Histogram 3	**Lyapunov**
Slope	WCOB D Fx 3	Wavelet Approx Kurtosis L1	Wavelet Approx Kurtosis L4	MFCC Kurtosis C1	TP Entropy	LyapunovExponentMean
SCBWCenter	WCOB D Fy 3	Wavelet Approx Entropy L1	Wavelet Approx Entropy L4	MFCC Median C1	TP Energy	LyapunovExponentStd
SCBWBandwidth	Hf1 3	Wavelet Approx Log Energy L1	Wavelet Approx Log Energy L4	MFCC Range C1	TP Skewness	LyapunovExponentMax
SpectralSkewness	Hf2 3	Wavelet Approx Max To Min Ratio L1	Wavelet Approx Max To Min Ratio L4	MFCC Entropy C1	TP Kurtosis	LyapunovExponentMin
SpectralKurtosis	Total Energy	Wavelet Approx Spectral Centroid L1	Wavelet Approx Spectral Centroid L4	MFCC Mean Abs Diff C1	TP Max Prob	**Recurrence**
PeakFrequency	Normalized Energy	Wavelet Approx Spectral Bandwidth L1	Wavelet Approx Spectral Bandwidth L4	Spectral Centroid C1	TP Ratio1	RecurrenceDeterminism
SpectralEnergy	Max Abs Bi	Wavelet Detail Mean L1	Wavelet Detail Mean L4	Spectral Bandwidth C1	TP Ratio2	**Amplitude Modulation**
SpectralEntropy	Mean Abs Bi	Wavelet Detail Std L1	Wavelet Detail Std L4	MFCC Mean C2	**EP**	AmplitudeModulationMean
ZeroCrossingRate	Entropy Skewness	Wavelet Detail Skewness L1	Wavelet Detail Skewness L4	MFCC Std C2	EP Energy	AmplitudeModulationStd
RMS	Entropy Kurtosis	Wavelet Detail Kurtosis L1	Wavelet Detail Kurtosis L4	MFCC Skewness C2	EP Mean Energy	AmplitudeModulationMax
SpectralFlatness	Symmetry Metric	Wavelet Detail Entropy L1	Wavelet Detail Entropy L4	MFCC Kurtosis C2	EP Max Energy	AmplitudeModulationMin
FM2MFreq1	Asymmetry Ratio	Wavelet Detail Log Energy L1	Wavelet Detail Log Energy L4	MFCC Median C2	EP Min Energy	AmplitudeModulationMedian
FM2MFreq2	Sym Mean	Wavelet Detail Max To Min Ratio L1	Wavelet Detail Max To Min Ratio L4	MFCC Range C2	EP Std Energy	**Miscellaneous**
FreqSkewness1	Sym Max	WaveletApproxSkewnessL3	Wavelet Detail Spectral Centroid L4	MFCC Entropy C2	**AVP**	DFA_ScalingExponent
FreqSkewness2	Sym Std	WaveletApproxKurtosisL3	Wavelet Detail Spectral Bandwidth L4	MFCC Mean Abs Diff C2	AVP Histogram 1	
SpectralCrest	Sym Var	WaveletApproxEntropyL3	**TQWT**	Spectral Centroid C2	AVP Histogram 2	
BandPower	Mean Value	WaveletApproxLogEnergyL3	TQWT	Spectral Bandwidth C2	AVP Mean	
**Bi-Spectrum Features**	Std Value	Wavelet Approx Entropy L2	SpectralEntropy	MFCC Mean C3	AVP Std	
En T Bi	Skewness Value	Wavelet Approx Log Energy L2	BandPowerLow	MFCC Std C3	AVP Max	
En T Bi D	Kurtosis Value	Wavelet Approx Max To Min Ratio L2	BandPowerMid	MFCC Skewness C3	AVP Min	
En F Bi	Range Value	Wavelet Approx Spectral Centroid L2	BandPowerHigh	MFCC Kurtosis C3	AVP Entropy	
En F Bi D	Energy Value	Wavelet Approx Spectral Bandwidth L2	**CQT**	MFCC Median C3	AVP Energy	
Mean Bi T F	Median Value	Wavelet Detail Mean L2	CQT Mean Power	MFCC Range C3	**DIR**	
Mean Bi D F	IQR Value	Wavelet Detail Std L2	CQT Std Power	MFCC Entropy C3	DIR Histogram 1	
Mean Bi T F G	Coef Variation	Wavelet Detail Skewness L2	CQT Skewness Power	MFCC Mean Abs Diff C3	DIR Histogram 2	
Mean Bi D F G	Region Area	Wavelet Detail Kurtosis L2	CQT Kurtosis Power	Spectral Centroid C3	DIR Histogram 3	
Mean Bi T F H	Bounding Box Area	Wavelet Detail Entropy L2	CQT Total Energy	Spectral Bandwidth C3	MAG Mean	
Mean Bi D F H	Aspect Ratio	Wavelet Detail Log Energy L2	CQT Temporal Centroid	MFCC Mean C4	MAG Std	
WCOB Tx	Centroid X	Wavelet Detail Max To Min Ratio L2	CQT Temporal Spread	MFCC Std C4	MAG Max	
WCOB Ty	Centroid Y	Wavelet Detail Spectral Centroid L2	CQT Spectral Centroid	MFCC Skewness C4	MAG Min	
WCOB Dx	Perimeter	Wavelet Detail Spectral Bandwidth L2	CQT Spectral Bandwidth	MFCC Kurtosis C4	DIR Entropy	
WCOB Dy	Compactness	Wavelet Approx Mean L3	CQT Spectral Flatness	MFCC Median C4	DIR Energy	
WCOB T Fx	Bounding Box Diagonal	Wavelet Approx Std L3	CQT Time Entropy	MFCC Range C4	**Noise Metrics**	
WCOB T Fy	Peak Value	Wavelet Approx Skewness L3	CQT Freq Entropy	MFCC Entropy C4	NoiseToHarmonicRatio	
WCOB D Fx	Frequency Centroid X	Wavelet Approx Kurtosis L3	CQT Gabor Energy Std	MFCC Mean Abs Diff C4	Shimmer	
WCOB D Fy	Frequency Centroid Y	Wavelet Approx Entropy L3	CQT Gabor Mean Mean	Spectral Centroid C4	Jitter	
H1	Frequency Bandwidth X	Wavelet Approx Log Energy L3	CQT Gabor Std Mean	Spectral Bandwidth C4	**PBP**	
H2	Frequency Bandwidth Y	Wavelet Approx Max To Min Ratio L3	CQT Gabor Skewness Mean	MFCC Mean C5	PBP Mean	
En F Bi D 1	Spectral Flux	Wavelet Approx Spectral Centroid L3	CQT Gabor Kurtosis Mean	MFCC Std C5	PBP Variance	
Mean Bi D F 1	Entropy Value	Wavelet Approx Spectral Bandwidth L3	CQT Freq Shifts Mean	MFCC Skewness C5	PBP Skewness	
WCOB D Fx 1	Texture Contrast	Wavelet Detail Mean L3	CQT Freq Shifts Std	MFCC Kurtosis C5	PBP Kurtosis	
WCOB D Fy 1	Texture Correlation	Wavelet Detail Std L3	CQT Freq Shifts Dynamic Range	MFCC Median C5	PBP Entropy	
Hf1 1	Texture Energy	Wavelet Detail Skewness L3	CQT Freq Intervals Mean	MFCC Range C5	LBP	
Hf2 1	Texture Homogeneity	Wavelet Detail Kurtosis L3	CQT Freq Intervals Std	MFCC Entropy C5	LBP Mean	
En F Bi D 2	Fractal Dimension	Wavelet Detail Entropy L3	CQT Bandwidth Mean	MFCC Mean Abs Diff C5	LBP Variance	
Mean Bi D F 2	Connected Components	Wavelet Detail Log Energy L3	CQT Bandwidth Std	Spectral Centroid C5	LBP Skewness	
WCOB D Fx 2	Euler Number	Wavelet Detail Max To Min Ratio L3	CQT Bandwidth Dynamic Range	Spectral Bandwidth C5	LBP Kurtosis	
WCOB D Fy 2	Num Holes	Wavelet Detail Spectral Centroid L3			LBP Entropy	

PSD: Power Spectral Density; Welch’s method: Welch’s method spectral estimation; CQT: Constant-Q Transform; MFCCs: Mel-Frequency Cepstral Coefficients; LBP: Local Binary Patterns; PBP: Probabilistic Binary Patterns; TP: Ternary Patterns; GBP: Gradient Binary Patterns; EP: Energy Patterns; AVP: Amplitude Variation Patterns; TQWT: Tunable Q-Factor Wavelet Transform; VMD: Variational Mode Decomposition; PSR: Phase Space Reconstruction; DFA: Detrended Fluctuation Analysis; NHR: Noise-to-Harmonics Ratio.

**Table A2:** Summary of selected features for each model.

Feature Number	Non-OSA vs. Mild-OSA	Non-OSA vs. Moderate-OSA	Non-OSA vs. Severe-OSA	Mild-OSA vs. Moderate-OSA	Mild-OSA vs. Severe-OSA	Moderate-OSA vs. Severe-OSA
1	MouthExpiration_Gap_163-296_SpectralEntropy	Average_Gap_45-329_SpectralSkewness	NoseInspiration_Gap_816-885_SpectralKurtosis	NoseExpiration_Gap_299-707_MeanPower	MouthExpiration_Gap_367-515_SCBW_Bandwidth	NoseExpiration_Gap_384-567_SpectralCrest
2	MouthExpiration_Gap_163-296_SpectralCrest	NoseInspiration_Gap_321-577_SpectralSkewness	NoseInspiration_Gap_816-885_FreqSkewness1	Mouth Expiration_BBox_229_712_22_26_iqrValue	Average_Average_BBoxes_EnFBiD_2	Average_BBox_584_362_21_35_numHoles
3	MouthExpiration_Gap_1375-1499_SpectralCrest	Average_BBox_548_370_24_29_entropyValue	NoseExpiration_Gap_986-1325_SCBW_Bandwidth	Mouth_Inspiration_FNMidFlow_MFCCMean_C1	Nose Expiration_BBox_787_244_15_15_rangeValue	Mouth_Inspiration_FNMidFlow_WaveletApproxEntropyL2
4	NoseInspiration_Gap_1066-1424_SCBW_Bandwidth	Mouth Inspiration_BBox_203_786_12_11_rangeValue	Average_BBox_527_294_11_18_textureEnergy	Nose_Expiration_FNMidFlow_WaveletApproxSkewnessL3	Average_Gap_73-307_SpectralCrest	Nose_Expiration_FNMidFlow_PeakCount
5	Mouth_Inspiration_FNMidFlow_MFCCMean_C1	Mouth_Expiration_FNMidFlow_WaveletApproxSpectralBandwidthL1	Average_BBox_526_336_15_18_centroidY	Mouth_Inspiration_FN_MFCCMean_C1	Nose Inspiration_BBox_766_223_15_15_medianValue	Mouth_Inspiration_FN_MFCCMean_C2
6	Mouth_Inspiration_FNMidFlow_MFCCMedian_C5	Mouth_Expiration_FNMidFlow_WaveletDetailEntropyL3	Nose Inspiration_BBox_0_0_22_23_aspectRatio	Mouth_Inspiration_FN_MFCCStd_C1	Nose Inspiration_BBox_220_207_581_615_peakValue	MouthExpiration_Gap_282-460_SCBW_Bandwidth
7	Nose_Expiration_FNMidFlow_WaveletApproxMeanL3	Mouth Inspiration_BBox_229_756_15_17_iqrValue	Mouth Inspiration_BBox_0_934_73_89_eulerNumber	Mouth_Inspiration_FN_SpectralCentroid_C1	Nose Inspiration_BBox_766_223_15_15_frequencyCentroidY	MouthInspiration_Gap_79-281_RMS
8	NoseExpiration_Gap_534-1003_BandPower	Nose Inspiration_BBox_533_433_14_13_coefVariation	Mouth Inspiration_BBox_74_955_16_16_meanValue	Mouth_Inspiration_FN_SpectralBandwidth_C1	Nose Inspiration_BBox_989_487_34_58_peakValue	NoseInspiration_Gap_327-532_MeanPower
9	NoseExpiration_Gap_1029-1329_SpectralSkewness	Nose Expiration_BBox_515_348_11_29_entropyValue	Mouth Inspiration_BBox_74_955_16_16_stdValue	Mouth_Inspiration_FN_MFCCKurtosis_C2	Nose Inspiration_BBox_989_487_34_58_frequencyCentroidY	NoseInspiration_Gap_327-532_SCBW_Bandwidth
10	Mouth Inspiration_BBox_10_480_22_39_numHoles	Average_FNMidFlow_DIR_Histogram_2	Mouth Expiration_Average_BBoxes_totalEnergy	Mouth_Expiration_FN_WaveletDetailSpectralCentroidL2	Nose Inspiration_BBox_989_487_34_58_spectralFlux	NoseInspiration_Gap_327-532_SpectralEnergy
11	Mouth Expiration_BBox_30_0_11_13_frequencyCentroidX	Average_FNMidFlow_AVP_Histogram_2	Mouth Expiration_Average_BBoxes_normalizedEnergy	Mouth_Expiration_FN_CQTBandwidthDynamicRange	Nose Inspiration_BBox_989_487_34_58_connectedComponents	NoseInspiration_Gap_327-532_BandPower
12	Mouth Expiration_BBox_504_333_51_42_kurtosisValue	Mouth_Inspiration_FNMidFlow_ZeroCrossing1	Mouth Expiration_Average_BBoxes_stdAbsBi	Mouth_Expiration_FN_MFCCStd_C1	Nose Inspiration_BBox_989_487_34_58_eulerNumber	Mouth Inspiration_BBox_290_464_45_80_regionArea
13	Mouth Expiration_BBox_504_333_51_42_boundingBoxArea	Mouth_Inspiration_FNMidFlow_WaveletDetailSpectralBandwidthL1	Mouth Expiration_Average_BBoxes_symStd	Mouth_Expiration_FN_SpectralCentroid_C1	Nose Inspiration_BBox_226_683_94_101_iqrValue	Nose Inspiration_BBox_367_371_287_304_iqrValue
14	Mouth Expiration_BBox_504_333_51_42_boundingBoxDiagonal	Mouth_Inspiration_FNMidFlow_WaveletDetailSpectralBandwidthL4	Mouth Expiration_BBox_588_259_169_356_perimeter	Mouth_Expiration_FN_MFCCSkewness_C2	Nose Inspiration_BBox_226_683_94_101_frequencyCentroidX	Nose Inspiration_BBox_367_371_287_304_textureHomogeneity
15	Mouth Expiration_BBox_977_478_46_82_numHoles	Mouth_Inspiration_FNMidFlow_MFCCMean_C2	Mouth Expiration_BBox_266_279_435_530_centroidY	Mouth_Expiration_FN_MFCCKurtosis_C2	Nose Inspiration_BBox_241_721_42_42_boundingBoxArea	Nose Inspiration_BBox_367_371_287_304_numHoles
16	Mouth Expiration_BBox_0_975_48_48_aspectRatio	Mouth_Inspiration_FNMidFlow_MFCCKurtosis_C5	Mouth Expiration_BBox_774_364_23_29_perimeter	Mouth_Expiration_FN_MFCCKurtosis_C3	Nose Inspiration_BBox_241_721_42_42_frequencyCentroidY	Nose Expiration_BBox_315_321_390_424_textureContrast
17	Mouth Expiration_BBox_0_975_48_48_textureContrast	Mouth_Inspiration_FNMidFlow_PBP_Skewness	Mouth Expiration_BBox_300_553_19_16_centroidX	Nose_Inspiration_FN_ZeroCrossing2	Nose Inspiration_BBox_243_745_14_14_textureEnergy	Nose Expiration_BBox_582_344_24_27_aspectRatio
18	Nose Inspiration_BBox_0_959_64_64_boundingBoxDiagonal	Mouth_Inspiration_FNMidFlow_TP_Histogram_1	Nose Inspiration_Average_BBoxes_EnFBiD_1	Nose_Inspiration_FN_WaveletApproxSkewnessL1	Nose Inspiration_BBox_243_745_14_14_eulerNumber	Nose Expiration_BBox_582_344_24_27_perimeter
19	Nose Inspiration_BBox_0_959_64_64_textureEnergy	Mouth_Inspiration_FNMidFlow_TP_MaxProb	Nose Inspiration_Average_BBoxes_MeanBiDF_1	Nose_Inspiration_FN_WaveletApproxSpectralCentroidL2	Nose Inspiration_BBox_223_765_15_16_stdValue	Average_FNMidFlow_WaveletDetailMaxToMinRatioL3
20	Average_FNMidFlow_WaveletApproxMaxToMinRatioL1	Mouth_Inspiration_FNMidFlow_EP_MaxEnergy	Nose Inspiration_Average_BBoxes_WCOBDFx_1	Nose_Inspiration_FN_WaveletDetailKurtosisL3	Nose Inspiration_BBox_223_765_15_16_entropyValue	Average_FNMidFlow_WaveletDetailKurtosisL4
21	Nose_Inspiration_FNMidFlow_HurstExponent	Mouth_Expiration_FNMidFlow_WaveletApproxKurtosisL1	Nose Inspiration_Average_BBoxes_WCOBDFy_1	Nose_Inspiration_FN_CQTStdPower	Nose Inspiration_BBox_202_787_14_17_entropyValue	Mouth_Inspiration_FNMidFlow_LyapunovExponentMean
22	Nose_Expiration_FN_KatzFD	Mouth_Expiration_FNMidFlow_WaveletApproxSkewnessL2	Nose Inspiration_Average_BBoxes_Hf1_1	Nose_Inspiration_FN_CQTSkewnessPower	Nose Inspiration_BBox_202_787_14_17_textureContrast	Mouth_Inspiration_FNMidFlow_BandPowerHigh
23	MouthInspiration_Gap_1217-1499_PeakFrequency	Mouth_Expiration_FNMidFlow_PBP_Kurtosis	Nose Inspiration_Average_BBoxes_Hf2_1	Nose_Inspiration_FN_CQTTemporalCentroid	Nose Inspiration_BBox_202_787_14_17_textureEnergy	Nose_Inspiration_FNMidFlow_WaveletApproxEntropyL1
24	Mouth Expiration_BBox_504_333_51_42_centroidY	Mouth_Expiration_FNMidFlow_PBP_Entropy	Nose Inspiration_Average_BBoxes_reserved1	Nose_Inspiration_FN_CQTSpectralCentroid	Nose Inspiration_BBox_0_989_39_34_stdValue	Nose_Inspiration_FNMidFlow_WaveletApproxEntropyL2
25	Mouth Expiration_BBox_504_333_51_42_peakValue	MouthInspiration_Gap_0-18_FM2MFreq1	Nose Inspiration_Average_BBoxes_EnFBiD_2	Nose_Inspiration_FN_CQTBandwidthDynamicRange	Nose Expiration_BBox_0_0_39_44_connectedComponents	Nose_Inspiration_FNMidFlow_WaveletDetailSpectralCentroidL3
26	Mouth Expiration_BBox_977_478_46_82_textureCorrelation	MouthInspiration_Gap_64-362_MeanPower	Nose Inspiration_Average_BBoxes_MeanBiDF_2	Nose_Inspiration_FN_MFCCSkewness_C1	Nose Expiration_BBox_465_0_66_46_medianValue	Nose_Expiration_FNMidFlow_WaveletApproxSkewnessL2
27	Nose Inspiration_BBox_0_0_38_38_rangeValue	MouthInspiration_Gap_64-362_RMS	Nose Inspiration_Average_BBoxes_WCOBDFx_2	Nose_Inspiration_FN_MFCCKurtosis_C1	Nose Expiration_BBox_465_0_66_46_iqrValue	Average_FN_AVP_Mean
28	Nose Inspiration_BBox_477_0_55_39_stdValue	MouthInspiration_Gap_64-362_FM2MFreq1	Nose Inspiration_Average_BBoxes_WCOBDFy_2	Nose_Inspiration_FN_SpectralBandwidth_C1	Nose Expiration_BBox_465_0_66_46_textureCorrelation	Mouth_Inspiration_FN_KatzFD
29	Nose Expiration_BBox_0_981_41_42_fractalDimension	MouthInspiration_Gap_64-362_BandPower	Nose Inspiration_Average_BBoxes_Hf1_2	Nose_Inspiration_FN_MFCCKurtosis_C2	Nose Expiration_BBox_465_0_66_46_textureEnergy	Mouth_Inspiration_FN_LyapunovExponentMax
30	Nose Expiration_BBox_495_986_47_37_stdValue	MouthInspiration_Gap_378-565_StandardDeviation	Nose Inspiration_Average_BBoxes_Hf2_2	Nose_Inspiration_FN_TP_Skewness	Nose Expiration_BBox_465_0_66_46_fractalDimension	Mouth_Expiration_FN_MFCCMedian_C3
31	Mouth_Expiration_FNMidFlow_MFCCMedian_C1	MouthInspiration_Gap_378-565_FM2MFreq1	Nose Inspiration_Average_BBoxes_reserved2	Nose_Expiration_FN_WaveletDetailSkewnessL2	Nose Expiration_BBox_465_0_66_46_connectedComponents	Nose_Inspiration_FN_WaveletDetailEntropyL3
32	Mouth_Expiration_FNMidFlow_PBP_Kurtosis	MouthInspiration_Gap_584-810_FM2MFreq1	Nose Inspiration_Average_BBoxes_bisEntropy	Nose_Expiration_FN_CQTMeanPower	Nose Expiration_BBox_873_128_24_23_centroidY	Nose_Inspiration_FN_CQTGaborEnergyMean
33	Mouth_Expiration_FNMidFlow_TP_Histogram_1	MouthInspiration_Gap_837-1499_FM2MFreq1	Nose Inspiration_BBox_140_140_733_744_spectralFlux	Nose_Expiration_FN_CQTSkewnessPower	Nose Expiration_BBox_873_128_24_23_compactness	Nose_Inspiration_FN_MFCCMedian_C3
34	Mouth_Expiration_FNMidFlow_TP_MaxProb	MouthExpiration_Gap_0-353_StandardDeviation	Nose Inspiration_BBox_140_140_733_744_connectedComponents	Nose_Expiration_FN_CQTSpectralDynamicsStd	Nose Expiration_BBox_841_162_21_21_energyValue	Average_Gap_306-496_Maximum
35	Mouth_Expiration_FNMidFlow_TP_Ratio1	MouthExpiration_Gap_815-1435_StandardDeviation	Nose Inspiration_BBox_782_151_70_73_skewnessValue	Mouth_Inspiration_FNMidFlow_ZeroCrossing1	Nose Expiration_BBox_787_244_15_15_frequencyCentroidX	Average_Gap_1282-1359_SCBW_Bandwidth
